# Preventive Saline Irrigation of the Bile Duct to Reduce the Rate of Residual Common Bile Duct Stones Without Intraductal Ultrasonography: A Systematic Review and Meta-Analysis

**DOI:** 10.7759/cureus.46720

**Published:** 2023-10-09

**Authors:** Usman A Akbar, Mounica Vorla, Ahmed Jamal Chaudhary, Maurish Fatima, Fnu Vikash, Sobaan Taj, Shaheryar Qazi, Zubair Khan

**Affiliations:** 1 Internal Medicine, Camden Clark Medical Center, West Virginia University, Parkersburg, USA; 2 Internal Medicine, Carle Foundation Hospital, Urbana, USA; 3 Internal Medicine, DMC Sinai Grace Hospital, Detroit, USA; 4 Medicine, King Edward Medical University, Lahore, PAK; 5 Gastroenterology and Hepatology, Montefiore Medical Center, Albert Einstein College of Medicine, Bronx, USA; 6 Internal Medicine, Jacobi Medical Center/Albert Einstein College of Medicine, Bronx, USA; 7 Internal Medicine, Jersey Shore University Medical Center, Neptune, USA; 8 Internal Medicine, Nishtar Medical University, Multan, PAK; 9 Gastroenterology, University of Texas at Houston, Houston, USA

**Keywords:** recurrent cholangitis, ultrasonography (usg), dilated common bile duct, saline flush-out, isotonic saline

## Abstract

Endoscopic retrograde cholangiopancreatography (ERCP) with endoscopic sphincterotomy (EST) has been proven efficacious in the removal of CBD stones. Even after endoscopic stone removal, recurring cholangitis due to a residual common bile duct (CBD) stone is prevalent in clinical practice with a residual recurrence rate of 4-24% after successful stone retrieval. This comprehensive study and meta-analysis aimed to determine if preventive saline irrigation of the bile duct (PSIB) reduces the amount of residual CBD stones. Through a comprehensive search of PubMed, EMBASE, Cochrane Library, and Web of Science until November 20, 2022, we identified 164 articles comparing the efficacy of PSIB and non-PSIB post-endoscopic CBD stone removal. After stringent selection, three studies were included for meta-analysis using ReviewManager (ReVman version 5.4.1; Cochrane, London, UK). Using a random effect (RE) model, we derived a pooled odds ratio (OR) with confidence interval (CI) (95%CI). A total of three studies have been included in the analysis. Out of which, two are randomized controlled trials (RCTs) and one is a non-randomized study. Out of 323 patients, 157 underwent PSIB after an endoscopic stone removal of CBD stones to reduce the residual of CBD stones, whereas 166 did not undergo saline irrigation (non-PSIB). In our analysis, PSIB significantly reduced the risk of residual stones (OR: 0.22, 95%CI: 0.11-0.45). However, there was no notable link between PSIB and post-irrigation cholangitis (OR: 1.08, 95%CI: 0.21-2.21). Although not statistically significant, PSIB showed a trend toward lowered risks of post-procedural pancreatitis (OR: 0.65), bleeding (OR: 0.68), and other complications (OR: 0.64). PSIB effectively reduces residual CBD stones after endoscopy, offering a cost-effective alternative to invasive procedures such as intraductal ultrasound (IDUS). However, larger RCTs are needed to validate its definitive role.

## Introduction and background

One of the most common gastrointestinal illnesses is cholelithiasis. According to a national survey conducted in Japan, 25.6% of cholelithiasis patients had common bile duct (CBD) stones, which frequently result in serious side effects, such as acute cholangitis, obstructive jaundice, and acute pancreatitis [1-3]. Residual CBD stones (RCBDS) are a known complication following endoscopic sphincterotomy (EST), occurring in approximately 20% of cases [4]. These stones can cause symptoms such as abdominal pain, jaundice, and pancreatitis and can increase the risk of developing cholangitis [5]. Several maneuvers have been performed to reduce the likelihood of recurrent stones, including the placement of a nasobiliary catheter immediately after the extraction of the stones following up with cholangiogram for residual CBDS detection, and there is also the involvement of intraductal ultrasonography (IDUS) detecting residual large or small fragments of stones [6,7].

Preventive saline irrigation of the bile duct (PSIB) is a procedure that involves the use of saline solution to flush out the bile duct and remove any remaining stones. It can be performed either percutaneously or endoscopically, depending on the location and size of the stones [8]. This simpler technique is believed to reduce the procedure costs and discomfort associated with IDUS or a follow-up cholangiogram [9].

Preventive saline irrigation usually involves flushing approximately 100 mL of normal saline and aspirated until a clear effluent is seen. Overall, PSIB appears to be a promising approach for reducing the rate of RCBDS and improving patient outcomes following cholecystectomy [8]. Endoscopic retrograde cholangiopancreatography (ERCP) with EST has been proven to be efficacious in the removal of CBD stones [10]. Even after endoscopic stone removal, recurrent cholangitis due to residual CBD stones is prevalent in clinical practice with a residual recurrence rate of 4-24% after successful stone retrieval [11]. This comprehensive systematic review and meta-analysis is aimed to determine if PSIB reduces the amount of residual CBD stones after ERCP.

## Review

Methods

**Figure 1:**
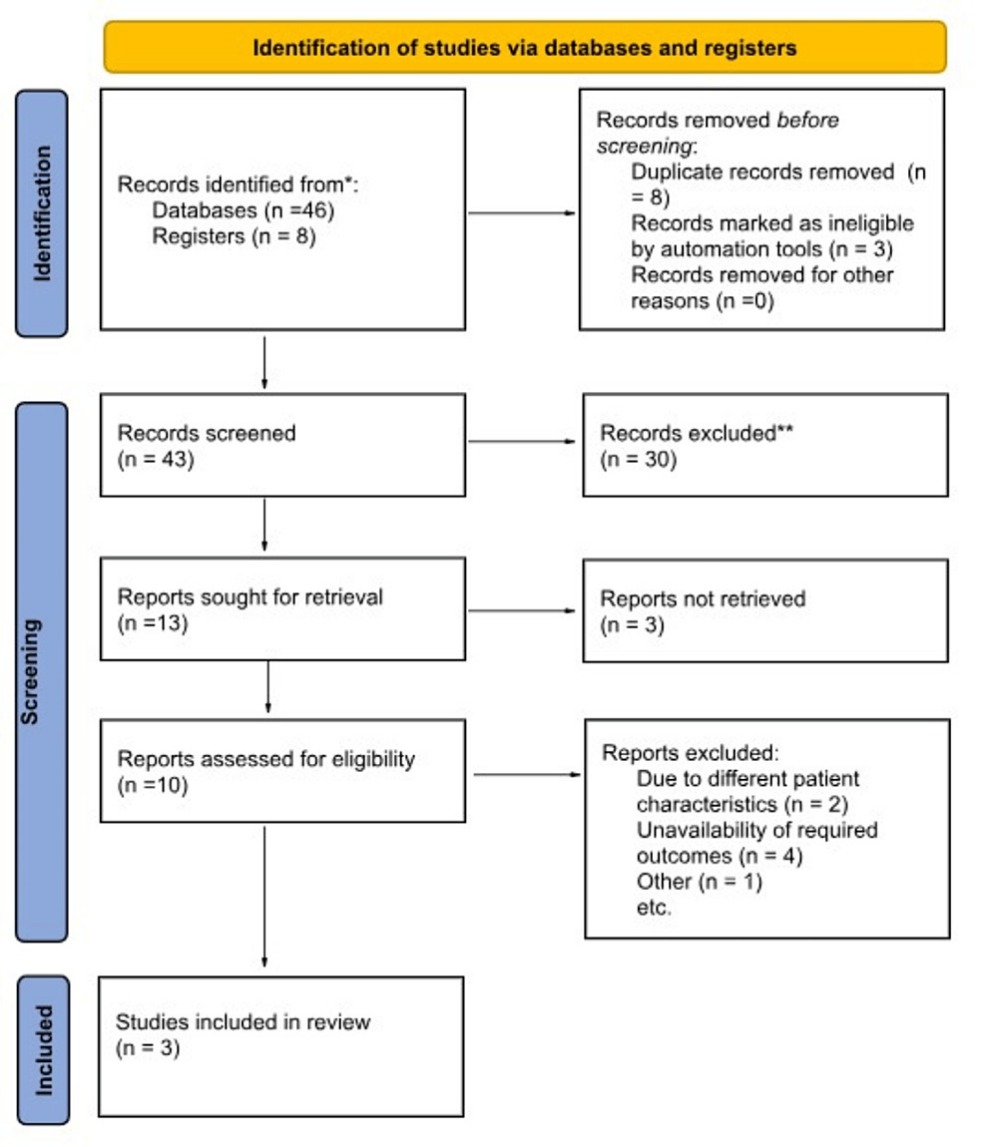
PRISMA flowchart

We performed this trial-level meta-analysis according to the Cochrane Collaboration guidelines and reported following the Preferred Reporting Items for Systematic Reviews and Meta-Analysis (PRISMA) [12,13] (Figure 1).

Data sources, searches, and study selection

We searched PubMed, EMBASE, Cochrane Library, and Web of Science from inception till November 20, 2022, for studies that compared the effectiveness of PSIB post ERCP in the reduction of residual CBD stones with non-PSIB, using broad search terms ("saline solution irrigation", "common bile ducts", "endoscopic retrograde pancreatography", "residual stones").

The pre-specified inclusion criteria were as follows: (1) randomized controlled trials (RCTs) and non-randomized studies (NRSs) comparing the outcomes of preventive saline irrigation in the development of bile duct stones after ERCP and (2) studies that report the outcomes of interest. We excluded case reports, case series, animal studies, systematic reviews, meta-analyses, and articles in foreign languages. Duplicates were eliminated, and the remaining articles underwent screening first at the title and abstract levels and subsequently at the full-text level (as illustrated in Figure 1). The study search and selection process was carried out independently by two authors (M.V and M.F), and any discrepancies or conflicts were resolved through discussion and mutual consensus.

Data extraction and risk of bias assessment

Data abstraction, assessment of data accuracy, risk of bias evaluation, and resolution of discrepancies were carried out independently by three reviewers (U.A.A, M.V, and M.F). The process included abstracting information related to trial and participant characteristics, such as age, gender, comorbidities, follow-up duration, inclusion/exclusion criteria, and the definition of primary endpoints. Additionally, the reviewers extracted data on the number of events and sample sizes. Any disagreements or inconsistencies were addressed through discussions or by referring to the original publications.

**Figure 2:**
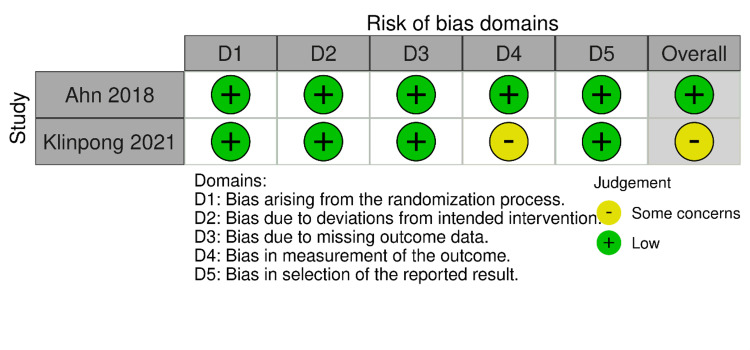
Bias assessment of randomized controlled trials (traffic light plots) Source: Refs. [14,15]

**Figure 3:**
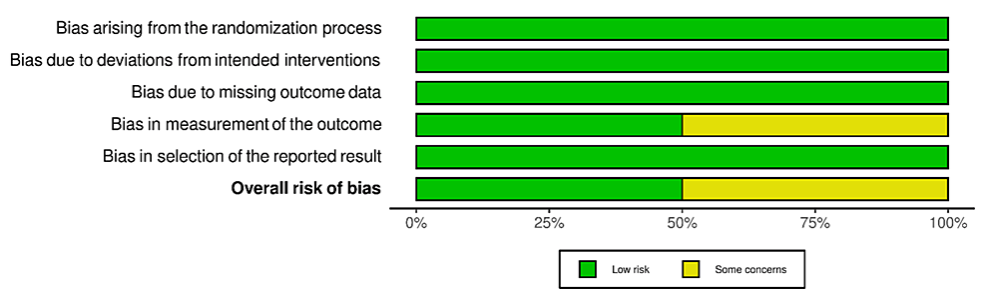
Bias assessment of randomized controlled trials (summary of plots) Source: Refs. [14,15]

**Figure 4:**
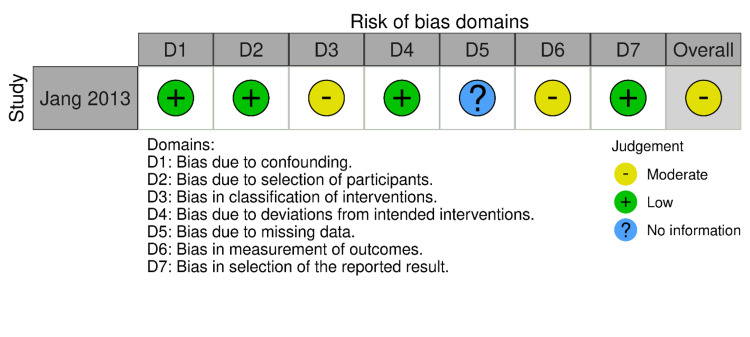
Bias assessment of non-randomized study (traffic light plots) Source: Ref. [8]

**Figure 5:**
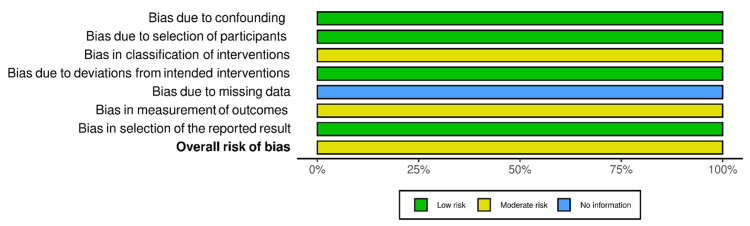
Bias assessment of non-randomized study (summary of plots) Source: Ref. [8]

RoB-2 and ROBINS-I were employed for the risk of bias assessment of included studies (Figures 2-5). We evaluated the risk of bias on a study level, considering various domains. These included bias stemming from the randomization process, bias arising from deviations in the intended interventions, bias due to missing outcome data, bias in the measurement of outcomes, and bias in the selection of reported results for RCTs. Additionally, for NRS, we assessed bias related to confounding, bias linked to the classification of participants, and bias associated with the classification of interventions.

Outcomes of interest

We primarily focused on residual CBD stones after endoscopic extraction of stones and post-procedural complications, such as post-procedure cholangitis, post-procedure pancreatitis, post-procedural bleeding, or any other complications.

Statistical analysis

Meta-analysis was performed via ReviewManager (ReVman version 5.4.1; Cochrane, London, UK). A Mantel-Haenszel random effect (RE) model was used to calculate the odds ratio (OR) with 95% confidence intervals (CIs). Funnel plots and Egger’s test were used to evaluate for publication bias. Additionally, we performed the chi-square test and I^2^ statistic to detect the presence of heterogeneity and quantify it, respectively. The I^2^ values were interpreted according to the Cochrane Handbook for Systematic Reviews of Interventions, and p < 0.10 was considered statistically significant for the chi-square test. For the primary outcome, we also conducted a sensitivity analysis by excluding NRSs with some concerns of bias due to the non-randomization process. A subgroup analysis based on the study design for the primary outcome was also performed.

Results

**Figure 6:**
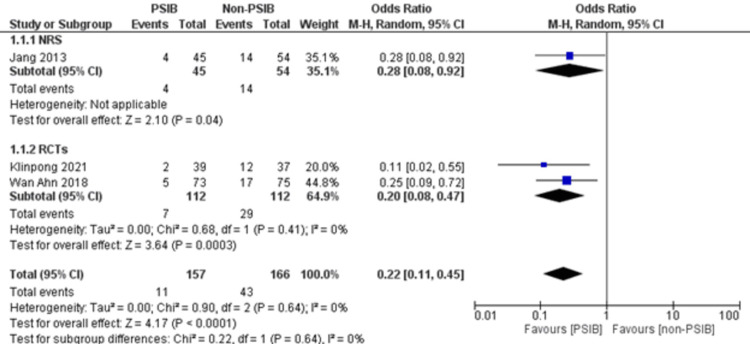
Forest plot comparing residual CBD stones in the PSIB and non-PSIB groups Sources: Refs. [8,14,15]

**Figure 7:**
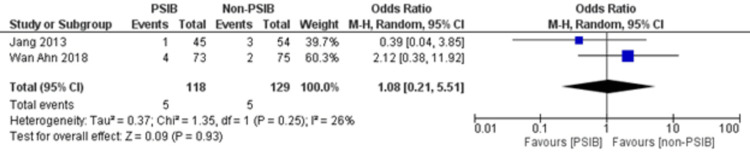
Post-procedural cholangitis Source: Refs. [8,14]

**Figure 8:**
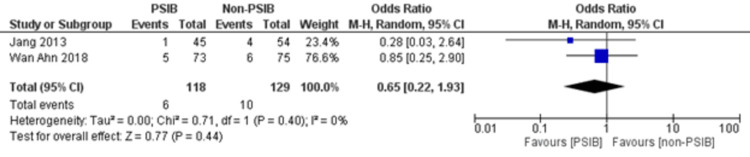
Forest plot comparing post-procedural pancreatitis between the PSIB and non-PSIB groups Source: Refs. [8,14]

**Figure 9:**
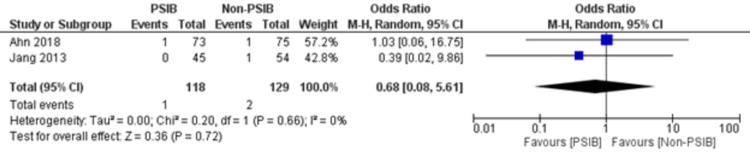
Forest plot comparing post-procedural bleeding between the PSIB and non-PSIB groups Source: Refs. [8,14]

**Figure 10:**
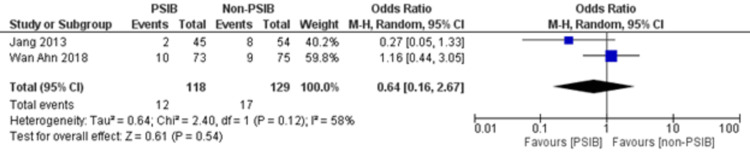
Forest plot comparing any other complication between the PSIB and non-PSIB groups Source: Refs. [8,14]

**Figure 11:**
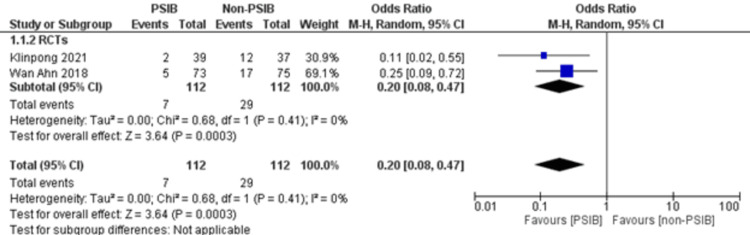
Sensitivity analysis of residual CBD stones after excluding NRSs Source: Refs. [14,15]

Two RCTs and one NRS were analyzed (Table 1). Out of 323 patients, 157 went through PSIB after endoscopic stone removal of CBD stones, whereas 166 did not undergo preventive saline irrigation (non-PSIB). A total of 173 (53.5%) patients were male, with 42% (n=136) patients having multiple stones and 14% (n=47) undergoing lithotripsy. The pooled analysis revealed a statistically significant decreased risk of the presence of residual stones with PSIB (OR: 0.22, 95%CI: 0.11-0.45, P<0.0001, I^2^=0%) (Figure 6). The subgroup analysis based on the study design found a similar association with the PSIB group in the subgroup of RCTs (OR: 0.20, 95%CI: 0.08-0.47, P=0.0003, I2=0%). The sensitivity analysis performed after excluding the NRS did not have much effect on the overall pooled OR (OR: 0.20; 95%CI: 0.08-0.47, P=0.0003, I^2^=0%). However, the pooled analysis of post-irrigation cholangitis demonstrated no significant association with either PSIB or non-PSIB (OR: 1.08, 95%CI: 0.21-2.21, P=0.93, I^2^=26%) (Figure 7). Though the results were not statistically significant, it demonstrated a decreased risk of post-procedural pancreatitis (OR: 0.65 95%CI: 0.22-1.93, P=0.44, I2=0%) (Figure 8), post-procedural bleeding (OR: 0.68, 95%CI: 0.08-5.61, P=0.72, I2=0%) (Figure 9) and other complications (OR: 0.64, 95%CI: 0.16-2.67, P=0.54, I2=58%) (Figure 10) with PSIB. Out of three studies, two were associated with a moderate risk of bias, and one study was found to have a low risk of bias. Figure 11 presents the sensitivity analysis performed after excluding the NRS.

**Table 1:** Baseline characteristics of the included studies

Author, Year	Population	Male/Female	Presence of multiple gall stones	Peri-ampullary diverticulum	Need for lithotripsy	Median follow-up period	Time for procedure	Residual stones in saline irrigation group	Residual stones in non-saline irrigation group	Post-irrigation cholangitis	Post-irrigation pancreatitis	Post-irrigation bleeding	Post-irrigation other complications
Ahn et al. 2018 [14]	n=148, PSIB=73, non-PSIB=75	male=81, female=67	PSIB=33, non-PSIB=30	PSIB=27, non-PSIB=31	PSIB=10, non-PSIB=10	12 m	PSIB=22, non-PSIB=19.2	5 of 73	17 of 75	PSIB=4, non-PSIB=2	PSIB=5, non-PSIB=6	PSIB=1, non-PSIB=1	PSIB=2, non-PSIB=8
Jang et al. 2013 [8]	n=99, PSIB=45, non-PSIB=54	male=59, female=40	PSIB=26, non-PSIB=34	PSIB=22, non-PSIB=17	PSIB=9, non-PSIB=13	25.2 m	PSIB=28.4, non-PSIB=21.2	4 of 45	14 of 54	PSIB=1, non-PSIB=3	PSIB=1, non-PSIB=4	PSIB=0, non-PSIB=1	PSIB=10, non-PSIB=9
Klinpong et al. 2021 [15]	n=76, PSIB=39, non-PSIB=37	male=33, female=43	Total=13	NA	Total=5	NA	NA	2 of 39	12 of 37	NA	NA	NA	NA

Discussion

This meta-analysis of two RCTs and one NRS consisting of 323 patients found that there is a decreased risk of the presence of residual bile stones in patients who went through PSIB after endoscopic stone removal of CBD as compared to the non-PSIB group. However, post-procedural complications, such as pancreatitis, bleeding, and any other complications, were not significantly associated with either group. Additionally, neither the PSIB group nor the non-PSIB group was found to be associated with post-irrigation cholangitis and post-procedural bleeding. There was an individualized decision regarding post-procedural cholangitis, which was specific to certain institutions’ policies.

Residual bile duct stones after ERCP have been reported to cause complications such as acute cholangitis [5]. Additionally, these stones can lead to long-term sequelae, such as persistent or recurrent cholangitis, chronic pancreatitis, and biliary stricture [16]. There is some evidence to suggest that the risk of post-procedural cholangitis may be higher with procedures that involve the placement of a naso-biliary catheter, such as post-sphincterotomy intrahepatic biliary drainage (PSID), due to the increased pressure in the CBD that can result from the placement of the catheter as reported by Ahn et al., but the pooled results did not report any significant association with either of the group [14]. This specific study discovered that irrigation with 100 mL of saline can flush residual stone fragments from the bile duct into the duodenum following stone removal. However, IDUS has a high diagnostic sensitivity and specificity for bile duct stones/debris. This method only produces indirect images of the debris.

The technique of using preventive saline for irrigation varied from one trial to another. For example, 100 mL of saline solution was injected through the catheter from the CBD and aspirated. When the retained stones or sludge were seen at this time, PSIB was performed again until a clear effluent was seen. A side-viewing duodenoscope (Olympus Optical Co., Ltd., Tokyo, Japan) was used. Stones were extracted using a basket and/or retrieval balloon, and mechanical lithotripsy was employed if necessary. In another study, a standard duodenoscope (JF 240, TJF 240, JF 260, or TJF 260) by Olympus was used. Biliary cannulation was attempted, and if difficult, alternative techniques such as precut sphincterotomy were used. Endoscopic sphincterotomy (EST) was performed, usually major EST, but limited EST was used in certain cases. Post-sphincterotomy balloon irrigation is done with 100 mL of sterile saline solution [8,14].

The proposed mechanism behind CDB stone recurrence involves the fragments of residual stones serving as a nidus for further CBD stone growth [17,18]. The placement of a naso-biliary catheter soon after the stone removal, a follow-up cholangiogram to detect residual CBD stones, and the visualization of residual CBD stones using IDUS are some of the maneuvers employed to decrease the possibility of retaining large or small residual stone fragments [19]. Currently, fluoroscopic cholangiographic imaging is the primary method for determining the successful removal of CBD stones [20]. Despite the absence of filling defects on cholangiography, IDUS studies have revealed the presence of residual biliary sludge within the bile duct. However, the disadvantages of these maneuvers include patient discomfort, the high cost of the procedure, and increased length of stay [7]. Therefore, simple procedures are needed that would reduce the occurrence of residual bile duct stones. Hence, PSIB without IDUS can serve as an alternative in reducing residual stone fragments and ultimately preventing a recurrence of cholangitis [19]. The reduced risk of post-procedural bleeding stems from several factors; saline washes away debris and clots, improving visibility and reducing tissue trauma. Saline's lubrication minimizes friction between instruments and the bile duct walls. Furthermore, its flushing action might reduce inflammation and the potential for bleeding by clearing irritants and residual stones.

In this review, PSIB has been associated with a statistically significant reduced incidence of residual bile duct stones without IDUS. This is consistent with findings from a similar study employing saline irrigation but using IDUS to detect residual stones [5]. Published data show that IDUS has been more sensitive than ERCP in detecting residual stones [2]. The difference in frequency of residual stone detection can be explained in the background of the sensitivity of IDUS detecting residual stones, which is higher than a CT or cholangiogram [21]. This also includes the time interval between stone removal and detection of residual stones. Furthermore, the rate of complications in our review did not differ between irrigation and the non-irrigation group, which can also be explained by shorter follow-up.

Among the included studies, in Jang et al. [8], endoscopy operators repeated the injection and aspiration with saline until the cholangiography revealed no residual stones or sludge. The technique took around seven minutes, whereas Ahn et al. [14] used 100 mL of sterile saline solution to perform PSIB, and it took three minutes longer than the non-PSIB group. Though the time required for PSIB was longer than the non-PSIB group, the technique is really useful and easy to perform in clinical practice [8,14,15]. Ahn et al. emphasized performing the procedure slowly in order to prevent excessive pressure build-up, leading to post-ERCP ascending cholangitis [14]. Additionally, no significant post-procedural mortality or morbidity was reported by individual studies, concluding that PSIB is a safe procedure [8,14].

To the best of our knowledge, this is the first meta-analysis to evaluate the role of PSIB in reducing the rate of residual bile stones after ERCP. The broad inclusion criteria enabled us to include NRSs in addition to RTCs and a better investigation of heterogeneity. The inclusion of studies from diverse settings, populations, and countries also adds to the strengths of this study.

One of the limitations of this meta-analysis is that two of the included studies used a follow-up of “six months” to define the presence of “residual stones” as it was difficult to confirm the presence of residual stones without IDUS [9,13], and, as a result, the residual stones may in fact be “true recurrent stones.” Other limitations include limited sample size and inclusion of an observational study that is, therefore, subject to biases and confounding that may have influenced our model estimates. A multi-center RCT with a larger sample size would be needed to further investigate the role of PSIB in reducing the risk of residual CBD stones. Additionally, due to no access to patient-level data, the meta-analysis was based on aggregate-level data.

## Conclusions

PSIB was associated with reduced residual CBD stones after endoscopic removal of stones. Additionally, there was no significant association with the risk of post-procedural complications, such as pancreatitis, bleeding, and other complications. The use of PSIB or non-PSIB did not influence the development of post-procedural cholangitis. Current methods for addressing this issue involve additional invasive procedures, such as the placement of naso-biliary catheters or IDUS, which can be uncomfortable for patients and costly. The findings of this review suggest that PSIB, without the need for IDUS, can serve as a feasible and effective alternative for reducing the occurrence of residual stone fragments and, consequently, preventing cholangitis recurrence. Future research, ideally in the form of multi-center RCTs with a larger sample size, is needed to further validate the role of PSIB in reducing the risk of residual CBD stones conclusively.
